# Modified Carbon Nanotube Paste Electrode for Voltammetric Determination of Carbidopa, Folic Acid, and Tryptophan

**DOI:** 10.1155/2012/305872

**Published:** 2012-05-15

**Authors:** Sakineh Esfandiari Baghbamidi, Hadi Beitollahi, Hassan Karimi-Maleh, Somayeh Soltani-Nejad, Vahhab Soltani-Nejad, Sara Roodsaz

**Affiliations:** ^1^Islamic Azad University, Bandar Abbas Branch, Bandar Abbas 7915893144, Iran; ^2^Environment Department, Research Institute of Environmental Sciences, International Center for Science, High Technology & Environmental Sciences, Kerman 7631133131, Iran; ^3^Department of Chemistry, Science and Research Branch, Islamic Azad University, Mazandaran 4816819195, Iran; ^4^Department of Chemistry, Islamic Azad University, North Tehran Branch, Tehran 1913674711, Iran

## Abstract

A simple and convenient method is described for voltammetric determination of carbidopa (CD), based on its electrochemical oxidation at a modified multiwall carbon nanotube paste electrode. Under optimized conditions, the proposed method exhibited acceptable analytical performances in terms of linearity (over the concentration range from 0.1 to 700.0 *μ*M), detection limit (65.0 nM), and reproducibility (RSD = 2.5%) for a solution containing CD. Also, square wave voltammetry (SWV) was used for simultaneous determination of CD, folic acid (FA), and tryptophan (TRP) at the modified electrode. To further validate its possible application, the method was used for the quantification of CD, FA, and TRP in urine samples.

## 1. Introduction

Electrochemical detection of analyte is a very elegant method in analytical chemistry [1]. The interest in developing electrochemical-sensing devices for use in environmental monitoring, clinical assays, or process control is growing rapidly. Electrochemical sensors satisfy many of the requirements for such tasks particularly owing to their inherent specificity, rapid response, sensitivity, and simplicity of preparation for the determination of organic molecules, including drugs and related molecules in pharmaceutical dosage forms and biological fluids [2, 3]. Carbon electrodes, especially glassy and paste electrodes, are widely used in electrochemical investigations [4–8].

 Electrochemical sensors based on carbon nanotubes (CNTs) represent a new and interesting alternative for quantification of different analytes. There are reports on the synthesis of multiwalled carbon nanotubes (MWCNTs) [9] and single-walled carbon nanotubes (SWCNTs) [10]. These materials have attracted enormous interest because of their unique structural, mechanical, electronic, and chemical properties. Some of these properties include high chemical and thermal stability, high elasticity, high tensile strength and, in some instances, metallic conductivity. The subtle electronic properties suggest that CNTs have the capability of promoting electron transfer reactions and improving sensitivity in electrochemistry, and thus they are widely used as electrodes [11–13]. CNT modified electrodes have been proved to have excellent electroanalytical properties, such as wide potential window, low background current, low detection limits, high sensitivities, reduction of over potentials, and resistance to surface fouling. There are reports that reveal that CNT modified electrodes have shown electrocatalytic behavior with excellent performance in the study of a number of biological species [14–18].

 Drug analysis is one of the important tools for drug quality control. Therefore, the development of simple, sensitive, rapid, and reliable method for the determination of drug is of great importance [19–21].

 Parkinson's disease victims show a significant depletion of dopamine in the brain. Since this neurotransmitter can not cross the blood-brain barrier into the central nervous system and it can not be employed to restore its normal level, levodopa (LD) (a precursor of dopamine) has been successfully used and is the most widely prescribed drug for the treatment of such patients [22, 23]. After its administration, LD is converted into dopamine via an enzymatic reaction catalyzed by dopa-decarboxylase. However, since the metabolism of LD is also extracerebral, several side effects of systemic dopamine can arise if LD is administered in high dosages. In order to achieve a better therapeutic effect and lower toxicity, carbidopa (CD) is administered in association with LD in pharmaceutical preparations, which contain 10–25% CD [24]. This catecholamine acts as an inhibitor for the decarboxylase activity. Hence, a combination of LD with CD leads to a control of the dopamine concentration at suitable levels, reducing the side effects and improving the efficiency of the therapy. Accordingly, the development of an analytical method is very important to control the content of these catecholamines in pharmaceuticals. Different techniques such as spectrophotometry, H-point standard addition, flow-batch, synchronous fluorescence spectrometry, and high-performance liquid chromatography have been employed for the determination of CD in pharmaceutical formulations [25–29]. Long analysis times, the use of organic solvents, and high costs are some of the drawbacks associated with these techniques. Voltammetry is considered as an important electrochemical technique utilized in electroanalytical chemistry because it provides low cost, sensitivity, precision, accuracy, simplicity, and rapidity [30, 31].

 Folic acid (FA) is a water-soluble vitamin and can act as coenzyme in the transfer and utilization of one-carbon groups and in the regeneration of methionine from homocysteine [32]. Deficiency of FA is a common cause of anaemia, and it is thought to increase the likelihood of heart attack and stroke. Many studies suggest that diminished folate status is associated with enhanced carcinogenesis as FA with vitamin B_12_ participates in the nucleotide synthesis, cell division, and gene expression [33]. Periconceptual supplementation of FA has been demonstrated to reduce significantly the incidence and reoccurrence of neural tube defects, such as spina bifida of women [34]. In January 1998, the US Food and Drug Administration introduced mandatory fortification of cereal grain products with FA at a concentration of 140 mg/100 g [35]. In the UK, the Department of Health proposed fortification of flour with FA at 240 mg/100 g [36]. Numerous methods for the measurement of FA are available. As FA is an electroactive component, some electrochemical methods have been reported for its determination. Comparing with other technologies, electrochemical method is more desirable because of its convenience and low cost [37–41].

 Tryptophan (2-amino-3-(1H-indol-3-yl)-propionic acid, TRP) is an essential amino acid for humans and a precursor for serotonin (a neurotransmitter), melatonin (a neurohormone), and niacin. It has been implicated as a possible cause of schizophrenia in people who cannot metabolize it properly. This compound is sometimes added to dietary, food products, and pharmaceutical formulas due to its scarce presence in vegetables [42]. Therefore, simple, sensitive and less expensive detection of TRP is of great interest. Therefore, various methods have been reported for the determination of TRP. Concentration of amino acids in biological samples is low; therefore it is necessary to use a highly sensitive method that provides determination of these analytes at subordinate concentrations. The electrochemical analytical technique is an attractive method due to its simplicity, low expense, high sensitivity, and possibility of miniaturization [43–46].

 In the present work, we describe the preparation of a new electrode composed of CNPE modified with ferrocene dicarboxylic acid (FCDCNPE) and investigate its performance for the electrocatalytic determination of CD in aqueous solutions. We also evaluate the analytical performance of the modified electrode for quantification of CD in the presence of FA and TRP.

## 2. Experimental

### 2.1. Apparatus and Chemicals

The electrochemical measurements were performed with an Autolab potentiostat/galvanostat (PGSTAT 302 N, Eco Chemie, the Netherlands). The experimental conditions were controlled with General Purpose Electrochemical System (GPES) software. A conventional three-electrode cell was used at 25 ± 1°C. An Ag/AgCl/KCl (3.0 M) electrode, a platinum wire, and the FCDCNPE were used as the reference, auxiliary, and working electrodes, respectively. A Metrohm 691 pH/ion meter was used for pH measurements.

All solutions were freshly prepared with double distilled water. CD, FA, TRP, and all other reagents were of analytical grade from Merck (Darmstadt, Germany). Graphite powder and paraffin oil (DC 350, density = 0.88 g cm^−3^) as the binding agent (both from Merck) were used for preparing the pastes. Multiwalled carbon nanotubes (purity more than 95%) with o.d. between 10 and 20 nm, i.d. between 5 and 10 nm, and tube length from 0.5 to 200 *μ*m were provided from Nanostructured & Amorphous Materials, Inc. The buffer solutions were prepared from orthophosphoric acid and its salts in the pH range of 2.0–11.0.

### 2.2. Preparation of the Electrodes

The FCDCNPEs were prepared by hand mixing 0.01 g of FCD with 0.89 g graphite powder and 0.1 g CNTs with a mortar and pestle. Then, ~0.7 mL of paraffin oil was added to the above mixture and mixed for 20 min until a uniformly wetted paste was obtained. The paste was then packed into the end of a glass tube (ca. 3.4 mm i.d. and 15 cm long). A copper wire inserted into the carbon paste provided the electrical contact. When necessary, a new surface was obtained by pushing an excess of the paste out of the tube and polishing with a weighing paper.

For comparison, FCD modified CPE electrode (FCDCPE) without CNTs, CNTs paste electrode (CNPE) without FCD, and unmodified CPE in the absence of both FCD and CNTs were also prepared in the same way.

### 2.3. Procedure of Urine Samples Preparation

Urine samples were stored in a refrigerator immediately after collection. Ten milliliters of the sample was centrifuged for 15 min at 2000 rpm. The supernatant was filtered out using a 0.45 *μ*m filter. Then, a different volume of the solution was transferred into a 10 mL volumetric flask and diluted to the mark with phosphate buffer (pH 5.0). The diluted urine sample was spiked with different amounts of CD, FA, and TRP.

## 3. Results and Discussion

### 3.1. Electrochemical Behavior of FCDCNPE

We have previously shown that a carbon paste electrode spiked with FCD is constructed by the incorporation of FCD in a graphite powder-paraffin oil matrix [47]. The experimental results show that well-defined and reproducible anodic and cathodic peaks were related to ferrocene dicarboxylic acid/ferricenium dicarboxylic acid (Fc/Fc^+^) redox system, which show a quasireversible behavior in an aqueous medium [48]. The electrode capability for the generation of a reproducible surface was examined by cyclic voltammetric data obtained in optimum solution, pH 5.0, from five separately prepared FCDCNPEs (Table 1). The calculated RSD for various parameters was accepted as the criterion for a satisfactory surface reproducibility (about 1–4%), which is virtually the same as that expected for the renewal or ordinary carbon paste surface [7, 12]. However, we regenerated the surface of FCDCNPE before each experiment according to our previous result [47].

 In addition, the long-term stability of the FCDCNPE was tested over a three-week period. When CVs were recorded after the modified electrode was stored in atmosphere at room temperature, the peak potential for CD oxidation was unchanged and the current signals showed less than 2.5% decrease relative to the initial response. The antifouling properties of the modified electrode toward CD oxidation and its oxidation products were investigated by recording the cyclic voltammograms of the modified electrode before and after use in the presence of CD. Cyclic voltammograms were recorded in the presence of CD after they have cycled the potential 20 times at a scan rate of 20 mV s^−1^. The peak potentials were unchanged and the currents decreased by less than 2.1%. Therefore, at the surface of FCDCNPE, not only does the sensitivity increase, but the fouling effect of the analyte and its oxidation product also decreases.

### 3.2. Influence of pH

The electrochemical behavior of CD is dependent on the pH value of the aqueous solution, whereas the electrochemical properties of Fc/Fc^+^ redox couple are independent of pH. Therefore, pH optimization of the solution seems to be necessary in order to obtain the electrocatalytic oxidation of CD. Thus, the electrochemical behavior of CD was studied in 0.1 M phosphate-buffered solutions (PBSs) in different pH values (2.0 < pH < 11.0) at the surface of FCDCNPE by cyclic voltammetry. It was found that the electrocatalytic oxidation of CD at the surface of FCDCNPE was more favored under acidic conditions than in neutral or basic medium. This appears as a gradual growth in the anodic peak current and a simultaneous decrease in the cathodic peak current in the cyclic voltammograms drawn at the surface of FCDCNPE. The variation of *I*_pa_ versus the variation of pH was studied. Results showed that the anodic peak current and the shifted potential value for electrooxidation of CD are high at pH 5.0. Thus, the pH 5.0 was chosen as the optimum pH for electrocatalysis of CD oxidation at the surface of FCDCNPE.

### 3.3. Electrocatalytic Oxidation of CD at an FCDCNPE

**Figure 1:**
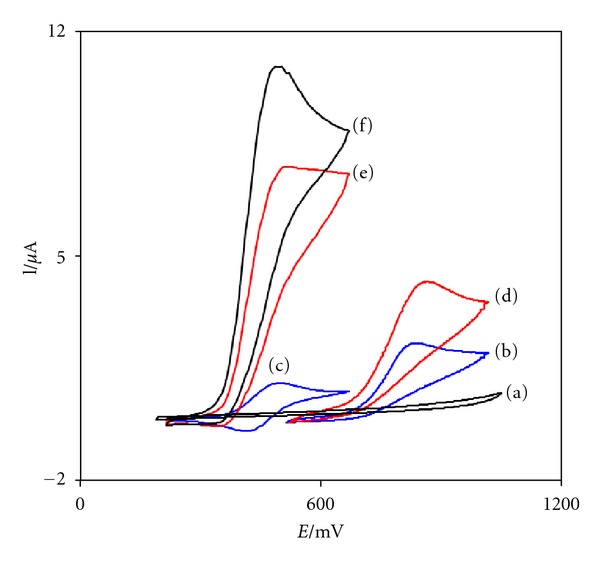
CVs of (a) unmodified CPE in 0.1 M PBS (pH 5.0), (b) unmodified CPE in 0.3 mM CD, (c) FCDCNPE in 0.1 M PBS, (d) CNPE in 0.3 mM CD, (e) FCDCPE in 0.3 mM CD, and (f) FCDCNPE in 0.3 mM CD. In all cases the scan rate was 20 mV s^−1^.

Figure 1 depicts the CV responses for the electrochemical oxidation of 0.3 mM CD at unmodified CPE (curve b), CNPE (curve d), FCDCPE (curve e), and FCDCNPE (curve f). As it is seen, while the anodic peak potential for CD oxidation at the CNPE and unmodified CPE is 830 and 890 mV, respectively, the corresponding potential at FCDCNPE and FCDCPE is *∼*500 mV. These results indicate that the peak potential for CD oxidation at the FCDCNPE and FCDCPE electrodes shifts by *∼*330 and 390 mV toward negative values compared to CNPE and unmodified CPE, respectively. However, FCDCNPE shows much higher anodic peak current for the oxidation of CD compared to FCDCPE, indicating that the combination of CNTs and the mediator (FCD) has significantly improved the performance of the electrode toward CD oxidation. In fact, FCDCNPE in the absence of CD exhibited a well-behaved redox reaction (Figure 1, curve c) in 0.1 M PBS (pH 5.0). However, there was a drastic increase in the anodic peak current in the presence of 0.3 mM CD (curve f), which can be related to the strong electrocatalytic effect of the FCDCNPE towards this compound [48].

**Figure 2:**
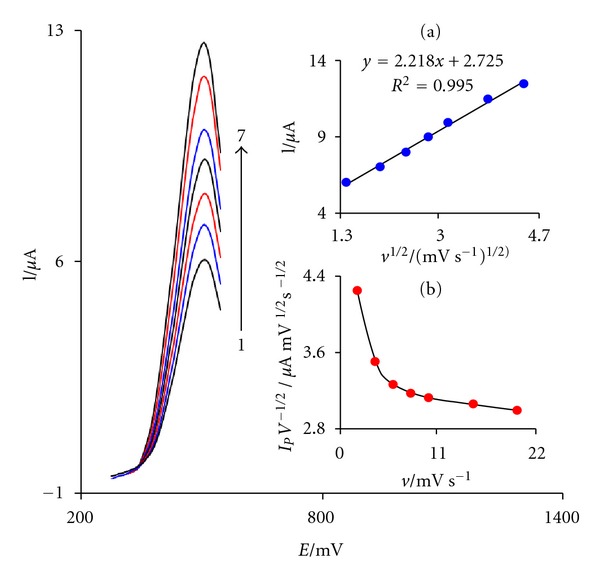
Linear sweep voltammograms of FCDCNPE in 0.1 M PBS (pH 5.0) containing 0.35 *μ*M CD at various scan rates; numbers 1–7 correspond to 2, 4, 6, 8, 10, 15, and 20 mV s^−1^, respectively. Insets: variation of (a) anodic peak current versus *ν* ^1/2^; (b) normalized current (*I*_*p*_/*ν* ^1/2^) versus *ν*.

 The effect of scan rate on the electrocatalytic oxidation of CD at the FCDCNPE was investigated by linear sweep voltammetry (LSV) (Figure 2). Also, a plot of peak height (*I*_*p*_) versus the square root of scan rate (*ν* ^1/2^) was found to be linear in the range of 2–20 mV s^−1^, suggesting that, at sufficient overpotential, the process is diffusion rather than surface controlled (Figure 2 (a)). A plot of the scan rate-normalized current (*I*_*p*_/*v* ^1/2^) versus scan rate (Figure 2 (b)) exhibits the characteristic shape typical of an EC process [48].

**Figure 3:**
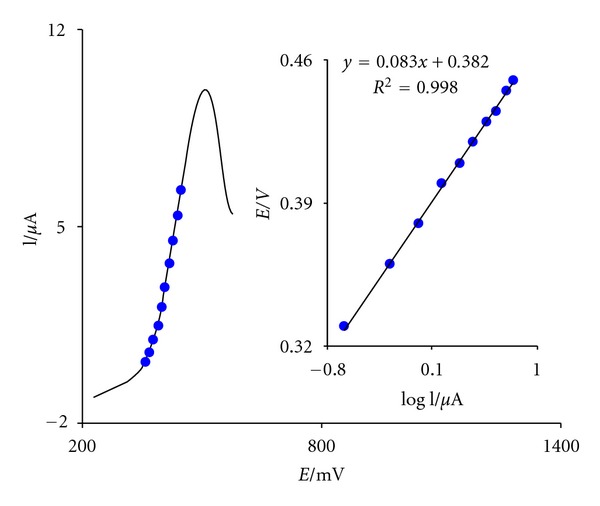
Linear sweep voltammogram (at 10 mV s^−1^) of FCDCNPE in 0.1 M PBS (pH 5.0) containing 0.15 mM CD. The points are the data used in the Tafel plot. The inset shows the Tafel plot derived from the linear sweep voltammogram.

Figure 3 shows a Tafel plot that was drawn from points of the Tafel region of the linear sweep voltammogram. The Tafel slope of 83.1 mV obtained in this case agrees well with the involvement of one electron in the rate determining step of the electrode process, assuming a charge transfer coefficient of *α* = 0.29.

### 3.4. Chronoamperometric Measurements

**Figure 4:**
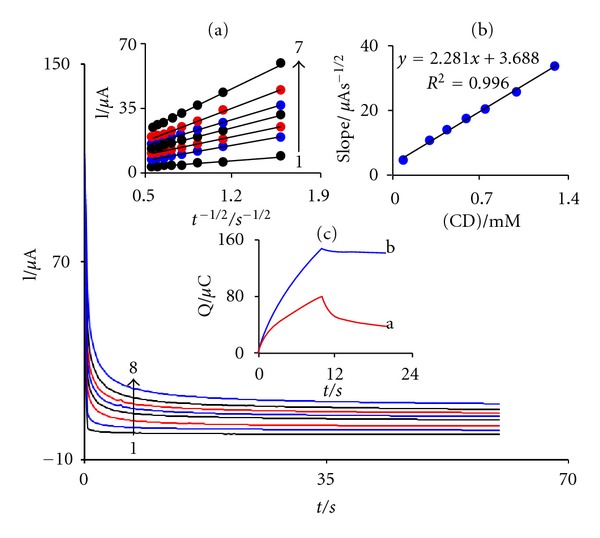
Chronoamperograms obtained at FCDCNPE in 0.1 M PBS (pH 5.0) for different concentrations of CD. The numbers 1–8 correspond to 0.0, 0.3, 0.1, 0.45, 0.6, 0.75, 1.0, and 1.3 mM of CD. Insets: (a) plots of *I* versus *t* ^−1/2  ^ obtained from chronoamperograms 2–8. (b) Plot of the slope of the straight lines against CD concentration and (c) FCDCNPE chronocolougrams in the absence (a) and presence of CD (b).

Chronoamperometric measurements of CD at FCDCNPE were carried out by setting the working electrode potential at 0.55 V versus Ag/AgCl/KCl (3.0 M) for the various concentrations of CD in PBS (pH 5.0) (Figure 4). For an electroactive material (CD in this case) with a diffusion coefficient of D, the current observed for the electrochemical reaction at the mass transport limited condition is described by the Cottrell equation [48]. Experimental plots of *I* versus *t* ^−1/2^ were employed, with the best fits for different concentrations of CD (Figure 4(a)). The slopes of the resulting straight lines were then plotted versus CD concentration (Figure 4(b)). From the resulting slope and the Cottrell equation the mean value of the D was found to be 5.4 × 10^−6 ^cm^2^/s.

Also, double potential step chronocoulometry, as well as other electrochemical methods, was employed for the investigation of electrode processes at FCDCNPE (Figure 4(c)). As can be seen, the forward and backward potential step chronocoulometry on the modified electrode in the blank buffered solution shows very symmetrical chronocolougrams with about an equal charge consumed for the oxidation and reduction of FCD in the CNPE (Figure 4(c), curve a). However, in the presence of CD the charge value associated with forward chronocoloumetry is significantly greater than that observed for backward chronocoloumetry (Figure 4(c), curve b). This behavior is typical of that expected for electrocatalysis at chemically modified electrodes [48].

### 3.5. Electrocatalytic Determination of CD

SWV method (with initial potential = 0.1 V, end potential = 0.6 v, step potential = 0.0195 v, amplitude = 0.045 v, and frequency = 10 Hz) was used to determine the concentration of CD. The plot of peak current versus CD concentration consisted of two linear segments with slopes of 0.0643 and 0.0138 *μ*A *μ*M^−1^ in the concentration ranges of 0.1 to 80.0 *μ*M and 80.0 to 750.0 *μ*M, respectively. The decrease in sensitivity (slope) of the second linear segment is likely due to kinetic limitation. The detection limit (3*σ*) of CD was found to be 65.0 nM. These values are comparable with values reported by other research groups for electrocatalytic oxidation of CD at the surface of chemically modified electrodes by other mediators (see Table 2).

### 3.6. Simultaneous Determination of CD, FA, and TRP

**Figure 5:**
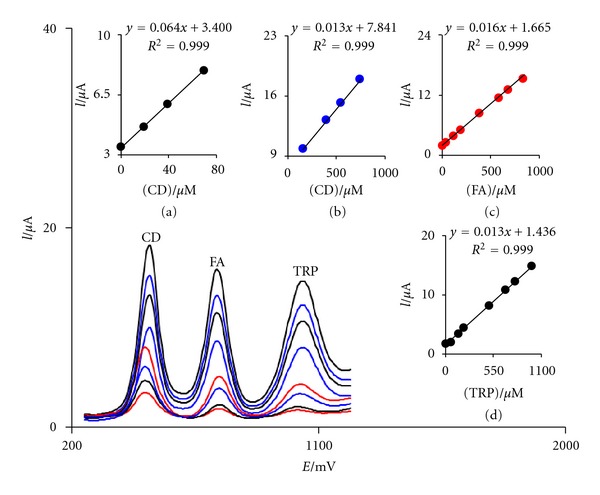
SWVs of FCDCNPE in 0.1 M PBS (pH 5.0) containing different concentrations of CD+FA+TRP in *μ*M, from inner to outer: 0.2 + 14.0+ 12.0, 20.0 + 50.0 + 40.0, 40.0 + 150.0 + 125.0, 70.0 + 200.0 + 200.0, 150.0 + 500.0 + 400.0, 400.0 + 700.0 + 600.0, 550.0 + 800.0 + 700.0, and 750.0 + 1000.0 + 850.0, respectively. Insets (a), (b), (c), and (d) are plots of *I*_*p*_ versus CD, FA, and TRP concentrations, respectively.

To our knowledge, there is no report on the simultaneous determination of CD, FA, and TRP using FCDCNPE. Therefore, the main objective of this study was to detect CD, FA, and TRP simultaneously using FCDCNPE. This was performed by simultaneously changing the concentrations of CD, FA, and TRP and recording the SWVs (with initial potential = 0.1 V, end potential = 1.2 V, step potential = 0.0195 V, amplitude = 0.045 V, and frequency = 10 Hz). The voltammetric results showed well-defined anodic peaks at potentials of 470, 740, and 1030 mV, corresponding to the oxidation of CD, FA, and TRP, respectively, indicating that simultaneous determination of these compounds is feasible at the FCDCNPE as shown in Figure 5.

### 3.7. Real Sample Analysis

To evaluate the applicability of the proposed method to real samples, it was applied to the determination of CD, FA, and TRP in urine samples. The CD, FA, and TRP contents were measured after sample preparation using the standard addition method. The results are given in Table 3.

## 4. Conclusions

In this study, a modified carbon nanotube paste electrode was fabricated for the voltammetric determination of CD. The enhancement in the oxidation current of CD and the negative shift of peak potential of CD at the modified electrode was observed. This newly developed method is sensitive, convenient, rapid, and suitable for determining CD. The modified electrode greatly catalyzed the electrooxidation reactions of CD, FA, and TRP, improving their oxidation peak separation. Thus, the large peak separations between CD, FA, and TRP allow their simultaneous analysis through square wave voltammetry technique. The proposed method could be applied to the determination of CD, FA and TRP in urine sample with quite promising results.

## Figures and Tables

**Table 1:** Cyclic voltammetric data obtained for constructed FCDCNPE in 0.1 M PBS (pH 5.0) at 10 mV s^−1^.

*E*_pa_ (V^a^)	*E*_pc_ (V)	*E*_1/2_ (V)	Δ*E*_*p*_ (V)	*I*_pa_ (*μ*A)	*I*_pc_ (*μ*A)
0.5 ± 1.2^b^	0.4 ± 1.3	0.4 ± 1.1	0.1 ± 1.2	1.0 ± 1.6	0.5 ± 1.8

^a^Versus Ag/AgCl/KCl (3.0 M) as reference electrode.

^b^All the “±” values are RSD% (*n* = 5).

**Table 2:** Comparison of the efficiency of some modified electrodes used in the electrocatalysis of LD.

Electrode	Modifier	Method	pH	Scan rate (mV/s)	Limit of detection (M)	Dynamic range (M)		Reference
Carbon paste	Ferrocene	Voltammetry	7.0	20	3.6 × 10^−6^	5.0 × 10^−6^	−6.0 × 10^−4^	[30]
Carbon paste	2, 2′-[1,2-ethanediylbis (nitriloethylidyne)]-bis-hydroquinone	Voltammetry	7.0	30	7.2 × 10^−6^	1.0 × 10^−5^	−6.0 × 10^−4^	[49]
Carbon paste	Ferrocene monocarboxylic acid	Voltammetry	7.0	10	2.9 × 10^−8^	7.0 × 10^−8^	−6.0 × 10^−4^	[50]
Carbon Nanotube paste	Ferrocene dicarboxylic acid	Voltammetry	5.0	20	6.5 × 10^−8^	1.0 × 10^−7^	−7.5 × 10^−4^	This work

**Table 3:** The application of FCDCNPE for simultaneous determination of CD, FA, and TRP in urine. All concentrations are in *μ*M (*n* = 5).

Spiked (*μ*M)			Found (*μ*M)			Recovery (%)			RSD (%)		
CD	FA	TRP	CD	FA	TRP	CD	FA	TRP	CD	FA	TRP
0	0	0	ND^a^	ND	ND	—	—	—	—	—	—
5.0	10.0	20.0	4.9	10.3	19.8	98.0	103.0	99.0	3.1	1.7	2.4
10.0	15.0	30.0	10.1	14.6	30.4	101.0	97.3	101.3	2.8	2.5	2.9
15.0	20.0	40.0	15.5	20.2	38.9	103.3	101.0	97.2	2.9	3.3	1.6
20.0	25.0	50.0	19.8	24.9	51.1	99.0	99.6	102.2	1.8	2.1	3.4

^a^ND: not detected.
